# Tuberculosis and Venous Thromboembolism: a case series

**DOI:** 10.1186/1757-1626-2-9333

**Published:** 2009-12-16

**Authors:** Ivone M Goncalves, Daniela Costa Alves, Aurora Carvalho, Maria do Ceu Brito, Fernando Calvario, Raquel Duarte

**Affiliations:** 1 Pneumology Department, Vila Nova de Gaia/Espinho Hospital Centre, Porto, Portugal; 2 Pneumology Department, São Marcos Hospital, Braga, Portugal; 3 Vila Nova de Gaia Pneumology Diagnostic Centre, Porto, Portugal

## Abstract

**Introduction:**

Tuberculosis remains an infectious disease with a high prevalence worldwide and represents a major public health issue. Although venous thromboembolism is a rare complication of this disease, it may be a potentially life-threatening event.

**Case series presentation:**

We report two cases of severe pulmonary tuberculosis associated with venous thromboembolism. A 38 year-old caucasian male that had a thromboembolic event as an unsual presentation form of tuberculosis and a 51 year-old caucasian male that developed deep venous thrombosis later in the course of the disease.

**Conclusion:**

An association between inflamation induced by tuberculosis and a hypercoagulable state has been described. Therefore, the occurence of deep venous thrombosis or pulmonary embolic episods, should be considered in patients with tuberculosis particulary during the first weeks of treatment. The physician's awarness of these phenomena is important to an early diagnostic suspicion and prompt treatment in order to prevent fatal outcomes.

## Introduction

Tuberculosis (TB) is one of the most prevalent infectious diseases in our country with an estimated incidence rate of 25.3 per 100.000 population in 2008 [1]. Worldwide, TB is responsible for more than 1.5 million deaths every year [1], with an estimated rate of 13.7 million prevalent cases of TB in 2007 (206 per 100.000 population) [2]. Therefore, despite recent progress, TB remains an important global public health problem [2], fact that should draw our attention to venous thromboembolism (VTE) as a possible complication of this disease.

Although deep venous thrombosis (DVT) is considered a rare event, it should be taken into consideration particularly in those with severe pulmonary or disseminated tuberculosis, as some authors correlate the risk of developing DVT increasing with the severity of the disease [3].

According to a retrospective analysis in a South African Hospital in the mid 1980s, *White et al*. stated that DVT rate was 3.4% within the first two weeks after initiation of therapy [4]. Recently, *Ambrosetti et al*., performed a nationwide prospective study comprising a routine evaluation of treatment outcomes in TB patients. This Italian group concluded that the prevalence of VTE was 0.6% in the first month of treatment, one third occurring in the first week. Furthermore, all cases except one, occurred in hospitalized patients [5].

Actually, VTE can be the presenting feature of TB [6], occur a few days after the diagnosis [7] or late in the course of the disease, even in patients on anti-tuberculosis treatment (ATT) [8].

Like other infectious diseases, TB can cause thrombosis by various mechanisms such as local invasion, venous compression [6] or by producing a transitory hypercoagulable state [9,10]. Recent studies have established a link between haemostatic changes and this prothrombotic phase, and have demonstrated that these can normalize with an adequate ATT [10].

Because VTE can be fatal, it is crucial to suspect it to perform an early diagnosis and initiate prompt treatment [3]. For this reason, patients that respond poorly to ATT, who have other predisposing factors and those in need of a prolonged stay in hospital, should be carefully monitored [10]. In some, prophylactic heparin should be prescribed and the use of venous catheters avoided [3].

We report two cases of severe pulmonary tuberculosis associated with VTE.

## Case report 1

A 38 year-old caucasian male, heavy smoker (40 pack/year), was presented to the emergency department (ED) with a painful swelling of the left lower limb of 15 days duration. He also complained of productive cough, anorexia and weight loss within the past 6 months. He was not on any medications and had no allergies. At that moment, he was an unemployed construction worker and a moderate alcohol consumer. General physical examination revealed a poorly built, malnourished man weighing 47 Kg. He was febrile (axilar temperature 38.3°C), taquicardic (pulse 102 bpm) and normotensive. Pulse oximetry (FiO2 21%) was 96% and chest auscultation revealed bilateral rhonchi. Cardiovascular and abdominal examination was normal. His left leg was swollen and tender to touch.

Arterial blood gas analysis (FiO_2 _= 21%) showed hypoxemia (pO_2 _= 89 mmHg). Laboratory findings on admission revealed a normal WBC count (7.9 G/mm^3^), a low hemoglobin level (11.3 g/dL), macrocytosis (MGV 106.2 fL), normal platelet count (397.000/mm^3^), hiponatremia (131 mEq/L) and elevated CRP (5.20 mg/dl) and d-dimer levels (5.51 ug/ml - normal < 0.5). Plasma fibrinogen level was also high (455 mg/dl - normal range 200-400).

**Figure 1:**
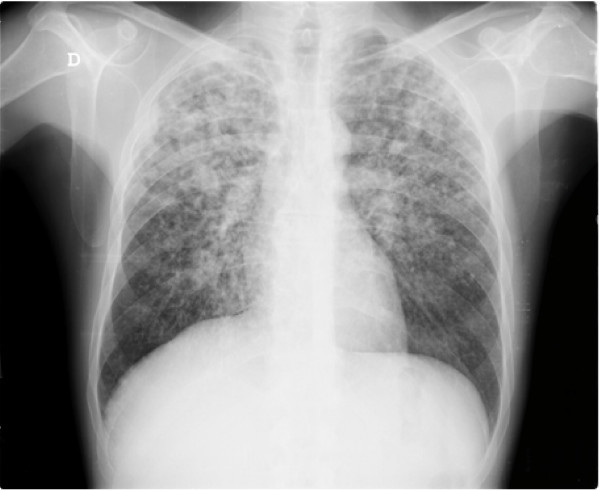
**Chest X-ray on admission**.

Chest X-ray demonstrated bilateral infiltrations and multiple cavitary lesions in both lungs (Figure 1). Deep venous thrombosis was confirmed by leg ultrasound.

**Figure 2:**
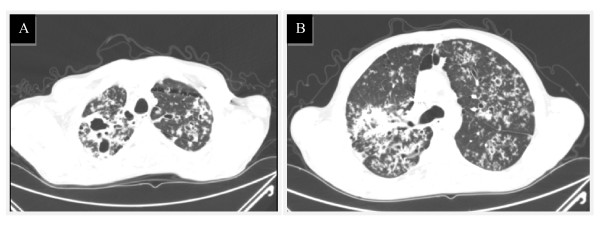
**Chest CT angiography on admission**. Multiple cavitary lesions in upper lobes and apical segments of lower lobes in communication with the bronquial tree (Figure 2A). Micronodules scattered in different pulmonary lobes with a "tree in bud" pattern. (bronquial disseminated tuberculosis). Hilar and paratracheal pericentimetric adenopathies were also identified (Figure 2B).

Chest CT angiography performed showed occlusion of distal segment of left pulmonary artery and right basal segmental branches, compatible with bilateral pulmonary embolism. There were also findings consistent with severe pulmonary tuberculosis (Figure 2A, B).

Mycobacterium tuberculosis (MT) infection was confirmed by culture on Lowenstein-Jensen agar and MT organisms were found to be sensitive to all first-line anti-tuberculosis drugs. Serologic testing for HIV, HBV and HCV were negative.

Standard ATT was initiated adjusted to weight: isoniazid (200 mg/day), rifampin (480 mg/day), pyrazinamid (1200 mg/day) and ethambutol (800 mg/day). The patient was treated with low-molecular-weight heparin (60 mg twice daily) and 3 days later, warfarin (5 mg/day) was started with a target International Normalized Ratio (INR) of 2.0-3.0.

On ATT his general state improved and he responded well to anticoagulation therapy. No adverse effects were seen. He was discharged 14 days after admission to continue follow-up at the Pneumology Diagnostic Centre. The patient has now finished two months on ATT regimen with isoniazid (200 mg/day) and rifampin (480 mg/day) with a good clinical response.

### Case 2

A 51 year-old male, ex-smoker, presented to the ED with a 3-week history of anorexia, productive cough with yellow sputum and weight loss. He also complained of night sweats and vomiting in the last 3 days. There was no prior history of other diseases but he had contacted in the last few months with a patient with TB. There was no history of alcohol abuse or sexual risk behaviors.

Clinical examination, revealed a thin, ill-appearing-man, weighing 40 kg. He was febrile (axilar temperature 38.4°C), hypotensive (BP 90/53 mmHg) and tachycardic (pulse 118 bpm). His respiratory rate was normal with an oxygen saturation of 98% (FiO_2 _= 21%). Examination of chest and abdomen was unremarkable. No peripheral edema was found.

Routine investigations revealed anemia (Hb 10.5 g/dl), leucocytosis (WBC 13.8 G/L) and a high platelet count (635.000/mm^3^). Other abnormal results included hiponatremia (129 mEq/L), high CRP (18.09 mg/dL) and fibrinogen levels (668 mg/dl). Arterial blood gas analysis (FiO_2 _21%) showed: pH 7.47; pO_2 _92 mmHg; pCO_2 _33.8 mmHg; HCO_3 _24.3 mmol/L; oxygen saturation 98.5%.

**Figure 3:**
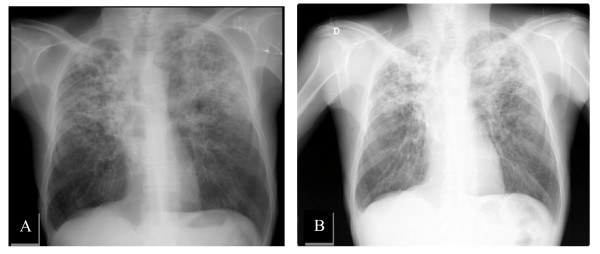
**Chest X-ray on admission (A)**. Chest X-ray 8 months later, showing exsudative lesions on both upper lobes (B).

Chest X-ray demonstrated a heterogeneous consolidation of both lung fields, mainly in the upper 2/3, consistent with tuberculosis (Figure 3A).

Sputum was found to contain acid-fast bacilli (> 36 bacilli/field) and MT subsequently cultured and found to be susceptible to all first line drugs. Serologic tests for HIV, HBV e HCV were negative.

ATT including isoniazid (250 mg/day), rifampicin (600 mg/day), pyrazinamid (1500 mg/day), ethambutol (1200 mg/day) and piridoxin (40 mg/day) was started. After two weeks in hospital, patient presented favorable response with improvement in constitutional symptoms and was discharged to continue ATT at the Diagnostic Pneumology Centre. He returned to the ED complaining of pain and swelling in his left limb 13 days later. Ultrasound revealed a femoropopliteal thrombosis. He was put on low-molecular-weight heparin (60 mg twice daily) and warfarin (5 mg/day) on the same day. Since therapeutic INR level was difficult to maintain with high doses (15 mg) of warfarin, acenocumarol was initiated.

Chest CT showed multiple confluent cavitary lesions mainly in the upper lobes, consolidation and micronodules, consistent with extensive pulmonary tuberculosis. A right hilar adenopathy (17 mm) and mediastinal adenopathies (some larger than 10 mm) were also observed. Abdominal and pelvic CT was performed and revealed homogeneous hepatomegaly and no focal parenchymal lesions. Tumor makers were negative. Since his bacteriological and radiological response (Figure 2B) to ATT was slow, he had to complete an 18-month therapy regimen.

## Discussion

Our cases show that VTE may complicate severe pulmonary tuberculosis and that these events occur at presentation or later in the course of the disease. *Robson et al*., found 35 patients with pulmonary TB and DVT. In 33 of them, DVT occurred 7 days after the diagnosis of TB, while only in two, DVT was the presenting feature [11]. Other reports also demonstrate that thrombotic phenomena in patients with pulmonary TB occur in other sites. These may include hepatic veins [8] and cerebral venous sinuses [12], which reinforces the link between these conditions.

Actually, tuberculosis is a disease with a wide variety of clinical presentations and recently, the association between inflammation, haemostatic changes and a hypercoagulable state has been established [9,10]. *Robson et al*. research study suggested that elevated plasma fibrinogen, impaired fibrinolysis coupled with decreased levels of antithrombin III and reactive thrombocytosis appeared to favour the development of DVT in pulmonary TB [11].

Similar observations were made by *Turken et al*. in a case-control study, regarding these haemostatic disturbances in 45 patients with active pulmonary TB. Moreover, it stated that these changes improved with ATT within 4 weeks [10]. On the other hand, there is also data supporting a relationship between this prothrombotic phase and a high frequency of antiphospholipid antibodies and protein S deficiency [3].

However, thrombosis can also result from venous compression by lymph nodes in ganglionar forms of TB, as retroperitoneal adenopathies may cause inferior vena cava thrombosis in the absence of any haemostatic abnormalities [6].

These haemostatic changes improve during the first month of ATT [10] and for this reason, it should be immediately started in addition to anticoagulant therapy. Frequently, a higher dose of warfarin is necessary to achieve therapeutic INR levels, because of rifampin effects on cytochrome P450 [13]. Additionally, this drug may also contribute to the hypercoagulable state by decreasing production and increasing clearance of anticoagulant hepatic proteins. Consequently, the initial phase of treatment may result in a higher risk for development of DVT [9].

## Conclusions

These clinical reports emphasize that patients with severe pulmonary tuberculosis are at risk of developing thromboembolic events. Therefore, these complications should be investigated, especially in those who do not improve on ATT, who have other predisposing factors or are hospitalized for long periods [10]. Prophylactic anticoagulant therapy should also be considered and the use of central venous catheters avoided in order to prevent venous thrombosis and its complications [3].

## Consent

Written informed consent was obtained from the patients for publication of these case series and any accompanying images. A copy of the written consent is available for review by the Editor-in-Chief of this Journal.

## Competing interests

The authors declare that they have no competing interests.

## Authors' contributions

IG and RD documented and prepared the draft, performed the literature search, revision of the bibiography and made substancial contributions to interpretation. FC, MCB and RD were the medical doctors who studied the patients, made substancial contributions to interpretation and helped in preparing the manuscript. DA made contributions to interpretation, performed the literature search and revision of the bibliography. RD and AC edited the manuscript. All authors have read and approved the final version of the manuscript and made substancial contributions to interpretation.
